# Specific use of start codons and cellular localization of splice variants of human phosphodiesterase 9A gene

**DOI:** 10.1186/1471-2199-7-39

**Published:** 2006-11-08

**Authors:** Carles Rentero, Pere Puigdomènech

**Affiliations:** 1 Departament de Genètica Molecular. Institut de Biologia Molecular de Barcelona. CSIC. Jordi Girona, 18. 08034 Barcelona. Spain; 2 Centre for Vascular Research. School of Medical Sciences. The University of New South Wales. Sydney. NSW 2052. Australia

## Abstract

**Background:**

Phosphodiesterases are an important protein family that catalyse the hydrolysis of cyclic nucleotide monophosphates (cAMP and cGMP), second intracellular messengers responsible for transducing a variety of extra-cellular signals. A number of different splice variants have been observed for the human phosphodiesterase 9A gene, a cGMP-specific high-affinity PDE. These mRNAs differ in the use of specific combinations of exons located at the 5' end of the gene while the 3' half, that codes for the catalytic domain of the protein, always has the same combination of exons. It was observed that to deduce the protein sequence with the catalytic domain from all the variants, at least two ATG start codons have to be used. Alternatively some variants code for shorter non-functional polypeptides.

**Results:**

In the present study, we expressed different splice variants of PDE9A in HeLa and Cos-1 cells with EGFP fluorescent protein in phase with the catalytic domain sequence in order to test the different start codon usage in each splice variant. It was found that at least two ATG start codons may be used and that the open reading frame that includes the catalytic domain may be translated. In addition the proteins produced from some of the splice variants are targeted to membrane ruffles and cellular vesicles while other variants appear to be cytoplasmic. A hypothesis about the functional meaning of these results is discussed.

**Conclusion:**

Our data suggest the utilization of two different start codons to produce a variety of different PDE9A proteins, allowing specific subcellular location of PDE9A splice variants.

## Background

Cyclic nucleotide monophosphates (cAMP and cGMP) are ubiquitous second intracellular messengers which play an essential role in the transduction of a variety of extracellular signals. The control of intracellular cyclic nucleotide levels is achieved by different enzymes, including cyclases that synthesize the cyclic nucleotides and catabolic enzymes such as cyclic nucleotide phosphodiesterases (PDEs). At least twenty one genes coding for cyclic nucleotide PDEs have been identified in mammals. They have been classified in 11 families and in humans they code for more than 60 different isoforms through alternative splicing [1-3].

The PDE9A gene encodes a cGMP-specific high-affinity PDE with widespread expression in most of the tissues examined [4-6]. The human PDE9A gene is located on 21q22.3, and has at least 22 exons spanning 122 kb. Because of its mapping position PDE9A is a possible candidate for genetic diseases mapped on 21q22.3, such as bipolar affective disorder. Furthermore, its overexpression might be involved in Down's syndrome [5]. For these reasons the expression in brain tissues of PDE9A, and also other PDEs, has been studied [7-9]. BAY 73–6691, a potent and selective PDE9A inhibitor, is currently under preclinical development for the treatment of Alzheimer's disease [10].

At least 20 different human PDE9A mRNA transcripts have been so far identified by EST analysis and sequencing of PCR amplification of cDNAs from different tissues, which are produced as a result of alternative splicing of 5' exons [4,5,11]. However, based on 5'RACE [8] and EST studies, there is only one alternative splice variant (equivalent to human PDE9A2 variant) that is present in mouse and rat. This result may indicate an important difference in the regulation of this gene in human and rodents at the level of splicing. These 20 different PDE9A splice variants could present different translation start codons to produce the functional protein. This mechanism has been described previously in other PDEs (PDE8A [12], PDE4D2 [13] and PDE7B [14]) but it has not yet been further studied. A complete review has been recently published summarizing the current efforts to understand the specific roles and functions of alternative splicing in metazoan [15].

The cyclic nucleotide-mediated pathways are regulated by a complex signalling system involving multienzyme families of adenylyl (AC) and guanylyl cyclases (GC), protein kinase A (PKA) and cGMP-dependent protein kinases (cGK), and cyclic nucleotide phosphodiesterases. One of the most important points of this signalling system is the cyclic nucleotide compartmentalization, which has been observed for cAMP in a number of cell types [16-18]. This can arise through the distinct intracellular localization of separate AC isoforms, the specific intracellular targeting of PDE isoforms, and the binding of PKA isoforms to specific anchoring proteins. For example, there are different PDE4 isoforms which have specific intracellular targeting [19,20], and interact with scaffolding proteins [21,22], due to the modification of specific N-terminal regions via alternatively spliced mRNAs in their 5' regions.

In this study we report the differential usage of PDE9A translation start sites depending on the splice variant, allowing the synthesis of a variety of PDE9A polypeptides that differ in their N-terminal regions. We also show the differential subcellular localization of certain PDE9A splice variants with different start codons that may have consequences for compartmentalization of cGMP signalling.

## Results and Discussion

### Different start codon usage of PDE9A splice variants

**Figure 1:**
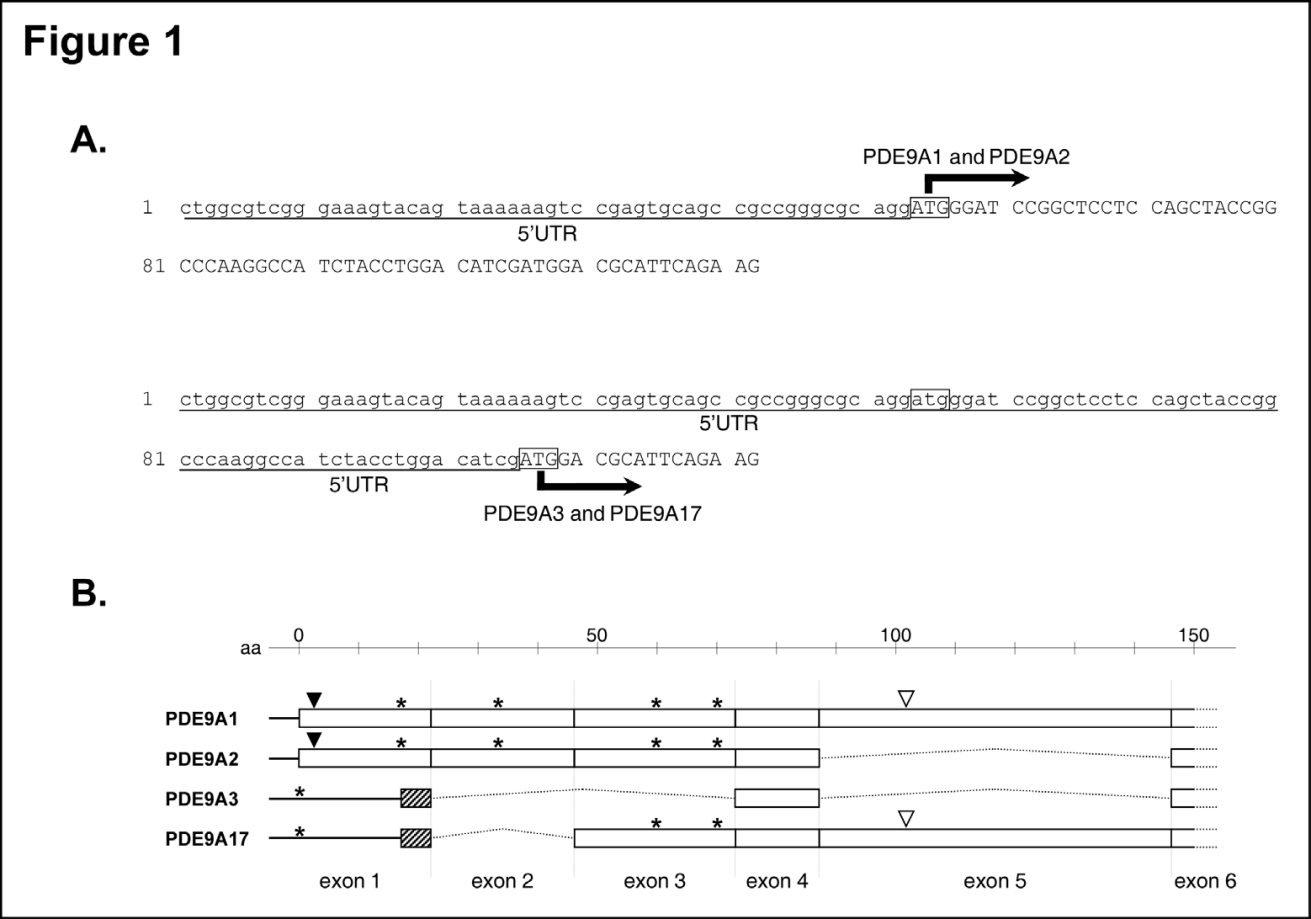
**Sequence and structure of the N-terminus region of human PDE9A splice variants**. (A) Sequences of the initial section of the PDE9A gene. The different ATGs are indicated by boxes and the predicted start codons for the different splice variants by wide arrows. (B) Structure of the 5' region of the human PDE9A splice variants. The exons are schematically illustrated by boxes, the 5'UTR by black lines, and the spliced exons by dotted lines. The amino acid sequence deduced from a shifted frame start codon is illustrated by hatched boxes, black triangles indicate the myristoylation motif location, white triangles indicate the pat7 motif location, and asterisks indicated other ATGs present in this sequence.

PDE9A has a large number of alternative splice variants that produce a number of mRNAs differing in their 5' region while the 3' domain that contains the region coding for the catalytic site of the enzyme is present in all cases. During this work, a new PDE9A splice variant called PDE9A21 (GenBank Accession Number AY701187) has been found by RT-PCR from human colon mRNA, which adds to the 20 splice variants previously described [4,5]. The sequence analysis of the predicted proteins indicated that if the open reading frame has to include, in phase, the PDE9A catalytic domain sequence in all the variants, a number of the different possible start codons (ATGs) could be used, depending on the splice variant [4]. According to the predicted sequence on the PDE9A1 and PDE9A2 splice variants (see Figure 1A), the first start codon in exon 1 is in phase with the complete ORF which has the catalytic domain. Using this ATG, other splice variants like PDE9A3 and PDE9A17 give two short proteins (61 and 78 amino acids respectively) due to early stop codons found in their ORF (in exons 6 and 5, respectively). However, there is a second ATG 52 bases downstream from the first one, also located on exon 1, in a shifted reading frame, which could be the start codon in PDE9A3 and PDE9A17, resulting in slightly shorter PDE9A proteins but having a complete catalytic domain. Figure 1B shows the schematic amino terminal representation of these splice variants. Therefore, this situation might lead either to the use of the first ATG, giving rise to a truncated protein, or to the use of a different downstream ATG that would produce a protein with a different N-terminus but that would include the catalytic domain. These four splice variants represent most of the PDE9A splice variants and would help us to clarify the usage of the different start codon in those transcripts.

In order to test these possibilities, cDNAs corresponding to the PDE9A1, PDE9A2, PDE9A3, and PDE9A17 splice variants were cloned in the pEGFP-N1 expression vector to create fusion proteins between the predicted ORF of the specific PDE9A isoform and that of EGFP fluorescent protein, and then they were transiently expressed in both Cos-1 and HeLa cells. The cDNAs were cloned with 53 nucleotides of the 5'-untranslated region (5'UTR) of PDE9A1 (from the theoretical ATG start codon of PDE9A1) and 105 nucleotides of the 5'UTR of PDE9A3 and PDE9A17 (from the theoretical ATG start codon of PDE9A3) (see Figure 1A). This region was obtained by 5' RACE on the longest cDNAs cloned [4]. These clones express the PDE9A splice variant followed by a GFP fluorescent protein in the C-terminal of the chimeric protein. All constructs were sequenced and the GFP sequence was, in all cases, in phase with the PDE9A catalytic domain. No changes in the sequence of the predicted PDE9A or GFP protein were observed.

**Figure 2:**
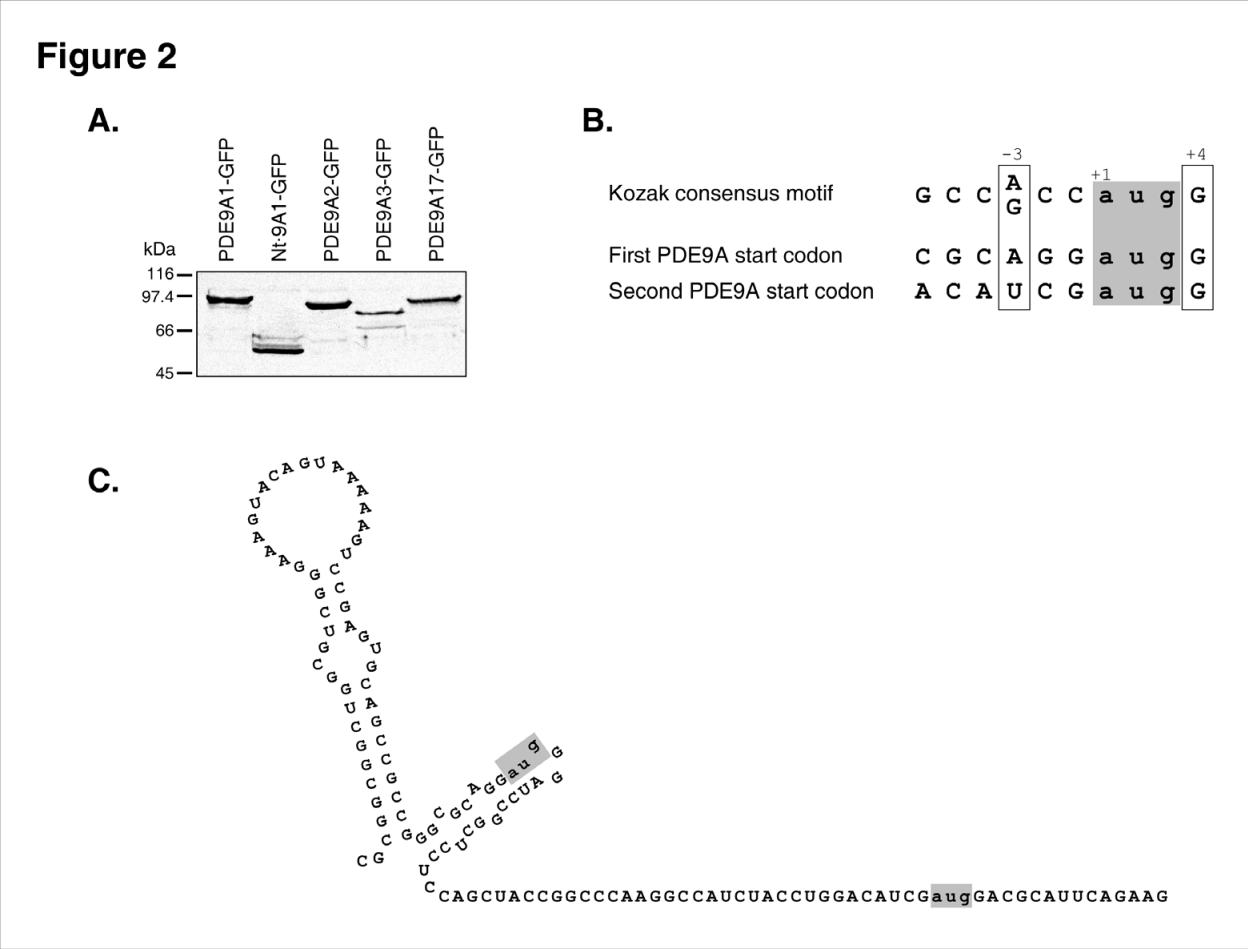
**Differential start codon usage in PDE9A splice variants**. (A) Western blot analysis of the expression of the chimeric PDE9A splice variants fused to GFP. GFP-fused proteins of PDE9A1 (lane 1, 96 kDa), the N-terminal region of PDE9A1 (lane 2, 62 kDa), PDE9A2 (lane 3, 89 kDa), PDE9A3 (lane 4, 81 kDa), and PDE9A17 (lane 5, 91 kDa) were produced in transfected Cos-1 cells, and were immunoblotted using a rabbit anti-GFP serum (1:1000). (B) Sequence comparison between Kozak consensus motif and PDE9A start codons, where the critical -3 and +4 positions are marked. (C) Schematic secondary structure representation of PDE9A exon 1 sequence. Start codons are emphasized by grey boxes.

The expression of the four PDE9A splice variants (PDEs 9A1, 9A2, 9A3 and 9A17) fused to GFP were tested in transiently transfected Cos-1 cells by immunoblotting analysis using both anti-GFP (see Figure 2A) and anti-PDE9A sera (data not shown). All splice variants (plus GFP) were detected with both antibodies and they show their predicted molecular mass (96.1, 88.6, 81.4, and 91 kDa respectively), revealing that both start codons were used by the cell to produce the chimeric PDE9A-GFP protein. The same results were obtained expressing these constructs in HeLa cells (data not shown). It may then be concluded that in the different splice variants of PDE9A, a variety of polypeptides with different N-terminal sequences but containing the catalytic domain are produced by the use of two different start codons present in exon 1.

Observing the mRNA initial sequence of PDE9A, a previously described hairpin structure [6] could be seen (Figure 2C), which could be the responsible element of a post-transcriptional regulation of PDE9A splice variants. There are some examples of proteins whose translation is affected by 5'UTR hairpin, such as ornithine decarboxylase translation inhibition, which is regulated together by its 5'UTR and enzymatic products [23]. Having a close look around exon 1 start codons sequence context and comparing them with Kozak consensus sequence (Figure 2B, and reviewed in [24]) we could see that the first start codon presents an optimal context for initiation of translation in mammals at sequence level, which could be affected by the secondary structure of the mRNA. On the other hand, the second start codon presents a weak or suboptimal context due to the pyrimidine on -3 position, but it might probably not be influenced by the inhibitory effect of the hairpin located upstream.

The different start codon usage in exon 1 reported here allows the differential translation of protein domains like the N-myristoylation motif present in the protein sequence region. A similar situation may be supposed to occur with the expression of splice variants of other PDEs such as PDE8A3, 4 and 5 splice variants [12], human PDE4D splice variants [13], and rat PDE7B2 splice variant [14] therefore producing a variety of proteins that differ in their N-terminus. Recent findings also show an increasing number of genes (like FGF2 [25]) with IRES elements (internal ribosome entry site elements) that regulate their translation by a balance between the cap-dependent and IRES-mediated expression to produce several proteins (reviewed in [26]).

### Intracellular location of PDE9A splice variants in transfected HeLa and Cos-1 cells

In a number of other PDE proteins it was found that the protein structure contains a phosphodiesterase catalytic domain in the C-terminus of the protein, and several different regions in the N-terminus produced by differential splicing of the exons. This situation provided a large number of different proteins which properties differ in the regulation of catalytic activity. They may include a cleavage site for caspase activities (rnPDE4A5) [27], a site for protein-protein interactions (SH3 domains in rnPDE4A5) [27], or consensus sequences for subcellular targeting (PDE3, rnPDE4A1A) [28,29].

Laser scanning confocal microscopy has been employed to define the intracellular localization of the proteins produced by the PDE9A1, PDE9A2, PDE9A3 and PDE9A17 splice variants. For this analysis, cDNAs corresponding to the different splice variants were transiently transfected in HeLa and Cos-1 cells with the GFP protein fused in the carboxy terminal region, and with the CFP protein fused in both the carboxy and amino terminal region of the predicted proteins, obtaining very similar results. The strategy chosen for the study has been to express the different PDE9A polypeptides in cultured cells. Expressing tagged-proteins in cases where no useful antibodies are available may be the only way to detect the location of the proteins within the cell, and fusing the tag in both the carboxy and amino terminal region maximize the reliability of this approximation.

**Figure 3:**
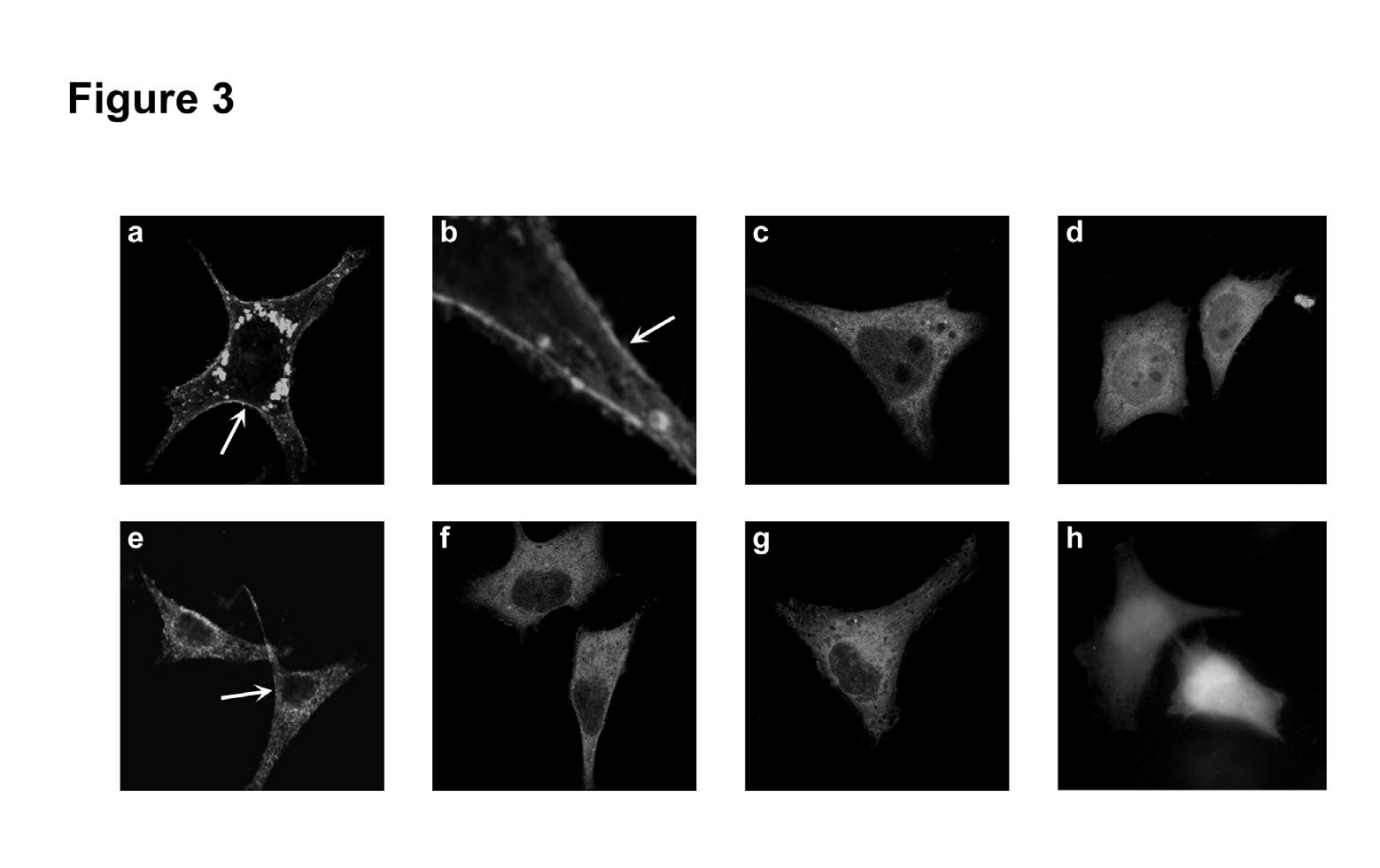
**Intracellular localization of GFP-chimeric PDE9A splice variants in transiently transfected HeLa cells**. All splice variant constructions were cloned with their 5'UTR region as described in Methods. Confocal analysis of: *a *and *b*, GFP-carboxy terminal-fused proteins of PDE9A1; *c*, N-terminal region of PDE9A1; *d*, C-terminal region of PDE9A; *e*, PDE9A2; *f*, PDE9A3; *g*, PDE9A17; and *h*, GFP control. Panel *b *is a detailed image of GFP-PDE9A1. White arrows indicate the membrane location of chimeric proteins.

**Figure 4:**
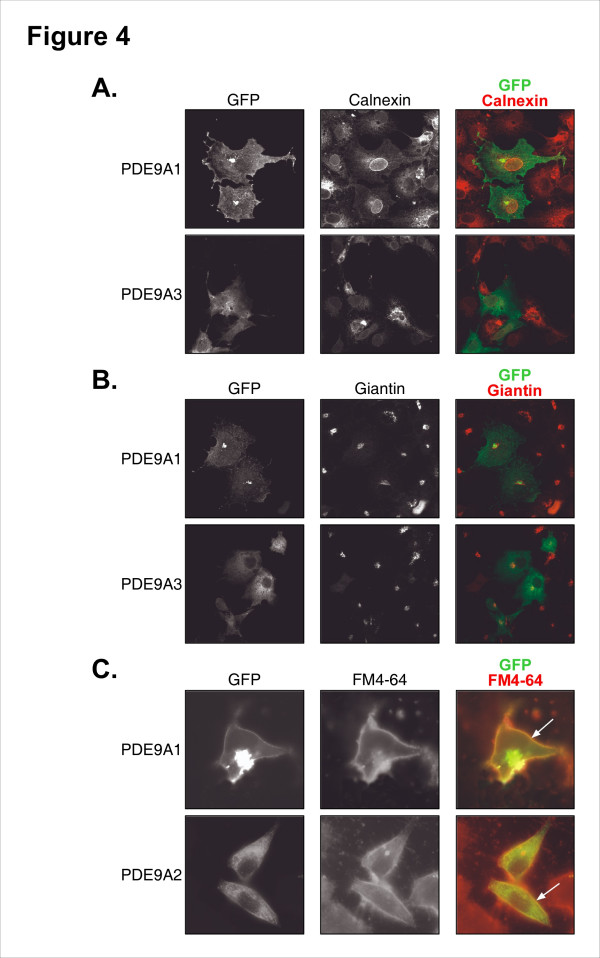
**Intracellular co-location of PDE9A1, PDE9A2 and PDE9A3**. Intracellular co-location of PDE9A1-, PDE9A2- and PDE9A3-GFP chimeric proteins with endoplasmic reticulum, Golgi apparatus and membrane markers in transiently transfected Cos-1 and HeLa cells. Confocal analysis of PDE9A1-GFP and PDE9A3-GFP with (A) calnexin (endoplasmic reticulum marker) and (B) giantin (Golgi apparatus marker). (C) Membrane location of PDE9A1- and PDE9A2-GFP chimeric proteins in transiently transfected HeLa cells by *in vivo *fluorescence microscopy. Black and white panels show the expression of GFP-carboxy terminal-fused proteins and the marker (calnexin, giantin and FM4-64) location, and the coloured image show the merged composition of both images. White arrows indicate the co-location of the chimeric proteins with the subcellular markers.

This study revealed that PDE9A1 is predominantly localized in the membrane ruffles at the cell margin, but it is also directed to a discrete perinuclear location (Figure 3a and 3b), Golgi apparatus (Figure 4B), intracellular vesicles, and weakly to endoplasmic reticulum (Figure 4A). In the detailed image (Figure 3b) the membrane and membrane ruffle association of PDE9A1 is visible as well as the location in vesicles. However, the amino terminal PDE9A1 construction (residues 1–304), lacking the C-terminal region that contains the catalytic domain, was not clearly expressed in the cell margin, but was distributed throughout the cell cytosol (Figure 3c). The carboxy terminal PDE9A construction, spanning the catalytic domain and the extreme carboxy terminal region of PDE9A (residues 271–593 of PDE9A1), was also distributed all around the cytosol (Figure 3d). A weak nuclear expression was detected in this construction, while PDE9A1 expression was excluded from the nucleus in all cases (Figure 3a and 3c). There was a high level of PDE9A2 in membrane ruffles and in the perinuclear region (Figure 3e). However, it was also present in other cell membrane regions. *In vivo *fluorescence microscopy of HeLa cells transiently transfected with PDE9A1-GFP and PDE9A2-GFP constructs and stained with FM4-64 has revealed the co-localization of PDE9A-GFP chimeric proteins and the fluorescent membrane tracer (Figure 4C). The reticular location of PDE9A2-GFP chimeric protein could also be observed in these images. The PDE9A3 and PDE9A17 splice variants, on the other hand, seem to have lost the targeting towards a cortical region having, instead, a cytosolic location (Figure 3f and 3g) and a weak colocalization with the endoplasmic reticulum (Figure 4A). In these cases there was a clear definition of the cell outline, which was missing from the control GFP expression (Figure 3h).

Different cellular location of the PDE9A splice variants may be regulated by the N-terminus region, which is the main change in the different splice variants. The analysis of this region shows that two putative domains (the pat7 and the bipartite motif) for nuclear localization in PDE9A are found in this region. The pat7 signal is present in splice variants 1 and 17 (exon 5, sequence PLRDRRV), and the bipartite signal is present in all splice variants studied (exon 19, sequence KKTDSLTSGATEKSRER). In contrast to our data, Wang and colleagues [30] found a pat7-dependent nuclear location or PDE9A1, whereas our chimeric constructions with the pat7 signal have never given a nuclear location. There is an N-myristoylation pattern (MGSGSSS) in the first amino acids of PDE9A1 and PDE9A2 (only in splice variants which utilize the first start codon in exon 1), which is often related to modifications leading to a membrane attachment. The presence of PDE9A1 in the Golgi apparatus is consistent with post-translational myristoylation, which is done in this cellular compartment.

The results obtained above indicate that both the amino and carboxy terminal regions take part in the regulation of the cellular localization of PDE9A, because only the complete sequence is found in the vicinity of the membrane region while a cytoplasmic location is observed for both N-terminal and C-terminal isolated regions. However, a recent structural study suggests that the N-terminal region of PDE9A does not significantly participate in the regulation of PDE activity [31]. Nevertheless only the longer splice variants show the specific pattern observed. It could then be possible that the cellular localization of PDE9A is regulated by differential splicing, which provides a specific combination of sequences, and post-translational modifications allowing a membrane targeting. These different types of modifications may explain the differences observed in studies that have used different cell types [30]. In this situation, the interaction with another protein or proteins may be important for the observed localization of the protein.

The presence of PDE9A splice variants in the vicinity of the membrane and in other subcellular locations may allow the regulation of cGMP degradation in cellular microdomains, which may be in accordance with cyclic nucleotide regulation due to multiple PDE proteins presence in each cell. These cellular microdomain locations, together with co-localization with other pathway signalling molecules probably contribute to both spatial and temporal regulation of specific cellular signal transduction.

## Conclusion

This study shows that the two start codons of PDE9A located on exon 1 could produce a variety of polypeptides with different N-terminal sequences but containing the catalytic domain. Moreover, these alternative splice variants present a different subcellular distribution, allowing the regulation of cGMP degradation in cellular microdomains contributing to a fine regulation of specific signal transduction.

## Methods

### Expression constructs

The GFP- and CFP-fusion constructs were prepared using pEGFP-N1, pECFP-N1 and pECFP-C1 (Clontech). Full-length PDE9A1, PDE9A2, PDE9A3 and PDE9A17 obtained by 5'RACE were PCR cloned into *Sac*I/*Sma*I sites of pEGFP-N1 and *Hin*dIII/*Sal*I sites of pECFP, resulting in PDE9A1 and PDE9A2 constructs with 53 nucleotides before their ATG start codon, and in PDE9A3 and PDE9A17 constructs with 105 nucleotides before their hypothetical ATG start codon. Amino terminal sequence of PDE9A1 (amino acid 1 to 304) and carboxy terminal sequence of PDE9A (amino acid 271–593 of PDE9A1) were PCR cloned into *Sac*I/*Sma*I sites of pEGFP1-N1, pECFP-N1 and pECFP-C1.

### Cell culture and transfection

Cos-1 (African green monkey kidney cell line) and HeLa cells (human cervix carcinoma cell line) were grown at 37°C under 5% CO_2 _in Dulbecco's Modified Eagle's Medium supplemented with 10% foetal bovine serum and 2 mM L-glutamine.

Cos-1 cells were seeded at about 33% of confluence in 100-mm diameter plates, and were transfected at approximately 50% confluence using the LipofectAMINE PLUS kit (Invitrogen) and 2 μg of each chimeric-DNA construct per plate. After 24 hours, cells were lysed with lysis buffer (50 mM HCl-Tris pH 8.0, 150 mM NaCl, 5 mM EDTA, 0.5% Nonidet P-40 and protease inhibitors).

For confocal analyses, HeLa and Cos-1 cells were plated out onto coverslips (12-mm diameter) at about 33% of confluence in 35-mm diameter plates, and were transfected at approximately 50% confluence using the LipofectAMINE PLUS kit and 1.5 μg of each chimeric-DNA construct per plate. After 24 hours, cells were fixed to the coverslips using paraformaldehyde 4%. The coverslips were mounted on glass slides with Mowiol (Calbiochem-Novabiochem). Calnexin and giantin were visualized using anti-calnexin [32] and anti-giantin [33] as first antibodies and goat anti-mouse (Alexa594) as secondary antibody (Molecular Probes). Samples were washed extensively and coverslips were mounted with Mowiol.

For fluorescence microscopy, HeLa and Cos-1 cells were plated out onto 22-mm diameter coverslips and transfected as described above. 24 hours after transfection, cell membranes were stained with the fluorescent lipophilic tracer FM4-64 (Molecular Probes) during 2 minutes in FM buffer (130 mM NaCl, 5 mM KCl, 2 mM CaCl_2_, 1 mM MgSO_4_, 0.8 mM Na_2_H(PO_4_), 0.2 mM NaH_2_(PO_4_), 25 mM glucose, and 20 mM HEPES). *In vivo *fluorescence microscopy was then performed.

### SDS-PAGE and western blotting

Rabbit polyclonal anti-PDE9A antibody was generated from the carboxy terminal fragment of PDE9A (amino acids 127 to 534). Briefly, this PDE9A C-terminal fragment was cloned in pET14b, expressed in *E. coli *BL21, and the His-tagged protein was purified by immobilised metal ion chromatography on Ni^2+^-nitriloacetic acid agarose (Ni-NTA). One microgram of purified recombinant protein was subcutaneously injected in presence of complete adjuvant (complete Freunds adjuvant) into New Zealand White rabbit, followed by two booster immunizations with incomplete adjuvant (incomplete Freunds adjuvant) 3 and 6 weeks after the priming immunization. After week 8 the animal was bled and serum prepared from whole blood.

For detection of chimeric PDE9A proteins by western blot, 10 μg of each lysate were boiled in Laemmly buffer [34], were separated by SDS/PAGE and transfered to nitrocellulose, followed by incubation with primary antibodies (anti-PDE9A and anti-GFP (Roche Applied Science)), the appropriated peroxidise-conjugated secondary antibodies (Jackson Immunoresearch) and ECL detection (Amersham Pharmacia).

### Confocal microscopy

Confocal microscopy was performed using a Leica TCS SP confocal laser scanning microscope fitted with spectrophotometers for emission band wavelength selection. An argon ion laser emitting at 488 and 458 nm was used to excite EGFP and ECFP respectively. During EGFP scanning, we used a triple-dichroic beam splitter (TD 488/543/633), the emission window set at 495–540 nm and a 63 × oil immersion objective. For ECFP scanning, a double-dichroic beam splitter (DD 458/514) was used, the emission window was set at 469–503 nm and a 63 × oil immersion objective was used. Serial optical slices of 0.5 μm were taken. Confocal image stacks were combined as x-y projection images.

## List of abbreviations

PDE, cyclic nucleotide phosphodiesterase; cAMP and cGMP, cyclic adenosine and guanosine monophosphate; EGFP and ECFP, enhanced green and cyan fluorescent proteins; 5'UTR, 5'-untranslated region; IRES, internal ribosome entry sites.

## Authors' contributions

PP conceived the experiment and co-wrote the paper. CR designed, carried out the experimental work and co-wrote the paper. Both authors read and approved the final manuscript.
